# Methodological and analytical limitations undermine reported outcomes of spinal DC stimulation in ALS

**DOI:** 10.3389/fneur.2025.1706131

**Published:** 2025-10-27

**Authors:** Morgan Highlander, Sherif Elbasiouny

**Affiliations:** ^1^Neuro Engineering, Rehabilitation, and Degeneration Lab, Department of Biomedical, Industrial, and Human Factors Engineering, College of Engineering and Computer Science, Wright State University, Dayton, OH, United States; ^2^Neuro Engineering, Rehabilitation, and Degeneration Lab, Department of Neuroscience, Cell Biology, and Physiology, College of Science and Mathematics, Wright State University, Dayton, OH, United States; ^3^Neuro Engineering, Rehabilitation, and Degeneration Lab, Boonshoft School of Medicine, Wright State University, Dayton, OH, United States

**Keywords:** electrical stimulation, direct current stimulation (DCS), amyotrophic lateral sclerosis (ALS), spinal stimulation, motoneuron, survival, SOD1-G93A

## 1 Introduction

Amyotrophic lateral sclerosis (ALS) is a fatal motoneuron disease that causes progressive loss of motor function and paralysis. While the exact causes of motoneuron degeneration remain unclear, abnormal electrical excitability of motoneurons (1) and altered cortical excitability (2) are consistent features (2). Treatments that normalize this excitability have shown promise in animal studies and influence current approved drugs (3, 4). However, existing pharmacological therapies provide only limited improvements in motor function and survival (5–8), highlighting the need for more effective treatment approaches.

Electroceuticals, which use targeted electrical stimulation rather than systemic drugs, offer a promising approach to regulate neuronal excitability. Therefore, a variety of electrical stimulation approaches have been studied in ALS including cortical (2, 9) and spinal (10–15) stimulations. So far, results are variable and the clinical impacts on disease progression have been limited (16).

Standing out from these prior studies, a recent study by Ahmed et al. examined the effects of anodal spinal DCS on the SOD1-G93A mouse model of ALS using unique electrode placements called “Multi-path DCS” (17). The treatment effects reported were profound, including prolonged motor function and an astounding improvement in animal survival of 74% (17). Such significant results warrant a deeper look into the study, which unfortunately uncovers several flaws that undermine the impact of results, as explained below. All figures referenced in the following text pertain to the Ahmed et al. study (17).

## 2 Opinion

### 2.1 Garnering false hope: 74% survival improvement is actually 5%

In preclinical models, the standard measure of survival is the interval from birth to when the animal reaches the humane endpoint (i.e., survival-from-birth) (10, 18–22), providing a direct measure of lifespan. In contrast, the study by Ahmed et al. quantifies survival as the duration between symptom onset and the humane endpoint (called survival-from-symptom onset), which more accurately reflects the progression of clinical symptoms rather than overall survival. While this measure may offer insights into disease trajectory, it does not constitute a valid proxy for lifespan extension, especially since symptom onset detection can be subjective and imprecise. Therefore, the reported 74% increase in survival-from-symptom-onset does not reflect a substantive improvement in total survival. The use of the term “survival” in this context is therefore misleading, as it inflates the perceived therapeutic benefit. Importantly, when survival-from-birth was directly assessed [Figure 2D in Ahmed et al. (17)], the observed increase in lifespan among stimulated animals was approximately 5%, indicating a considerably more modest effect than initially suggested.

### 2.2 Misapplication of statistical tests compromises survival outcome reliability

Although both parametric and non-parametric statistical analyses of survival data are presented, neither approach is supported by appropriate validation of underlying assumptions. Specifically, independent *t*-tests were applied to both survival-from-birth and survival-from-symptom onset datasets [Figures 2B, D in Ahmed et al. (17)], yet no assessment of normality was reported. If the data are not normally distributed, the application of the *t*-test is inappropriate and renders the resulting *p*-values unreliable. Conversely, for the non-parametric analyses [Figures 2A, C in Ahmed et al. (17)], the Breslow test was employed rather than the more commonly accepted Log-Rank test. The Breslow test assigns greater weight to early events, whereas the Log-Rank test distributes weight uniformly across time points (23). The authors provide no rationale for prioritizing early deaths over later ones, raising concerns about the suitability of the Breslow test. This issue is particularly relevant given the data in Figure 2D in Ahmed et al. (17), which indicate that two animals in the non-stimulated group died markedly earlier than the rest, and also earlier than expected based on published survival data for this model (24). It is plausible that the reported 74% survival improvement is disproportionately influenced by these outliers. Additionally, given their early death, it is unclear whether these animals survived long enough past their symptom onset to receive a single sham stimulation or died before receiving any sham stimulation. Collectively, the absence of normality testing and the questionable use of weighting in non-parametric tests undermine the statistical validity of the survival-from-birth and survival-from-symptom onset findings, reducing confidence in the reported treatment effects.

### 2.3 Delayed and unvalidated symptom onset detection inflates survival metrics

Another significant concern regarding the “survival-from-symptom onset” metric reported by the authors is the bias introduced by delayed and unvalidated detection of symptom onset. Accurate identification of symptom onset is critical in this context, as the 74% increase in survival-from-onset is calculated from this event. However, the ages at which symptom onset was detected in both groups were not reported in the study, making it impossible to assess the validity or consistency of this measurement.

Notably, while the survival-from-birth for the non-stimulated group (129.7 ± 2.3 days) aligns closely with published data for the SOD1-G93A high copy mouse model (130.2 ± 11.2 days) (24), the reported survival-from-onset (12.4 ± 1.4 days) is markedly shorter than the 61–62 days commonly reported in literature (19). This substantial discrepancy suggests that symptom onset in the current study was detected significantly later than in prior reports. This result is not unexpected given that the scoring method used to determine symptom onset was originally developed for assessing spasticity following spinal cord injury (25), and has not been validated against established ALS onset markers such as tremor onset (19), rotarod performance (20), neuroscrore (21), treadmill endurance (20), or hanging-wire test (20). While the authors attempt to justify their approach by providing correlation data between grid-walking scores and both hanging-wire performance and hind paw grip strength [Supplementary Figure S4 in Ahmed et al. (17)], this analysis is limited to a single, unspecified time point during what is described as the early disease stage. Given the evidence of delayed onset detection, this likely represents a mid- to late-stage disease state, thereby reducing the relevance of the correlation to true disease onset. Furthermore, observing that symptomatic animals perform worse than controls at an arbitrary time point does not validate the scoring method as a sensitive or reliable marker of disease onset. Still further, the description of the scoring methodology lacks clarity. Although the authors cite a previous publication to explain their approach, the referenced study used a different scoring system (range: 50–150, derived from ladder wheel performance) (25), whereas the current study reports a 0–6 score based on grid-walking behavior (25), suggesting a substantial methodological divergence without adequate justification or validation.

In sum, the method used for detecting symptom onset appears both delayed and unvalidated, likely inflating the measured improvement in survival-from-onset and casting doubt on the reliability of the reported treatment effect.

### 2.4 Supplementary data contradict reported survival benefits and reveal statistical inconsistencies

Additional evidence undermining the reported survival benefit arises from data presented in Supplementary Figure S3 in Ahmed et al. (17), in which stimulation was administered to a separate cohort beginning at 60 days of age and continuing until the detected onset of symptoms. Given the delayed symptom detection employed in this study, this treatment window likely extended well-beyond the actual onset of motor symptoms as typically defined in the SOD1-G93A model. Notably, this survival analysis revealed no significant treatment effect, which aligns more closely with previous studies that have reported only modest benefits of trans-spinal direct current stimulation in ALS models (10).

Further concerns emerge from the statistical approach used in Supplementary Figure S3 in Ahmed et al. (17). Unlike the analyses in Figure 2 in Ahmed et al. (17), which employed the Breslow test, a log-rank test was used here without justification for the change in methodology. This is particularly problematic because the Kaplan-Meier survival curves in Supplementary Figure S3 in Ahmed et al. (17) cross, violating the proportional hazards assumption required for valid application of the log-rank test (10). In such cases, alternative methods such as time-dependent Cox models or weighted tests are recommended. Paradoxically, while the log-rank test was inappropriately applied to crossed survival curves in Supplementary Figure S3 in Ahmed et al. (17), it was omitted from Figure 2 in Ahmed et al. (17), where it would have been appropriate given the absence of curve crossings.

Taken together, these inconsistencies in statistical methodology and the lack of significant survival effects in the more conservatively analyzed cohort raise substantial doubts regarding the validity of the survival findings reported by Ahmed et al. The survival analyses, as conducted, fail to adhere to established best practices, thereby undermining confidence in the study's conclusions.

### 2.5 Motor function preservation claim not supported by statistical evidence

The purported “preserved motor function” (17) effect claimed in the Ahmed et al. study is not statistically supported. Grid walking is the sole measure of motor function reported [Figure 2E in Ahmed et al. (17)], and it is from a selected subgroup of eight of the 48 animals in the survival study, with no indication of how or why these eight animals were selected, raising concerns about potential sampling bias. Furthermore, no statistical tests were applied to these data, and the scoring method itself was not adequately validated against established motor function assessments for the SOD1-G93A mouse model, such as rotarod, hanging-wire, treadmill, or grip strength assays. Compounding these concerns is the limited interpretability of the data since motor function over age is not presented. Rather, motor function scores are shown only as a function of relative days post-onset. As a result, it is not possible to contextualize the grid walking data with respect to normative disease progression curves or benchmark functional decline reported in prior studies.

Collectively, the absence of statistical analysis, lack of methodological transparency, and use of a non-validated and isolated functional measure provide insufficient evidence to support the claim of treatment-induced motor function preservation.

### 2.6 Outlier-driven and poorly controlled histological analyses fail to support treatment claims

The immunohistochemical analyses presented in Figures 7–10 in Ahmed et al. (17) suffer from significant methodological and reporting deficiencies, particularly regarding sampling procedures and stereological rigor. The study does not specify how the reported 2–3 tissue sections per animal were selected, nor does it clarify the number of z-stacks, fields of view, or individual cells analyzed. Additionally, the disease stage at which tissue was collected is not indicated.

For motoneuron quantification, analysis was conducted using Adobe software; however, there is no mention of size-based inclusion criteria or identification of choline acetyltransferase to confirm motoneuron identity and exclude non-motoneuronal cells. Moreover, the use of raw fluorescence intensity as a proxy for protein expression is methodologically problematic without stringent safeguards against experimental bias (26–29). The study fails to report whether critical controls—such as batch-randomized tissue processing, consistent antibody labeling conditions, and standardized imaging acquisition—were employed to prevent technical drift and batch effects. Vague statements such as “z-stack was used to ensure accuracy” and “same light set-up” offer little technical clarity or assurance of reproducibility.

In Figure 7 in Ahmed et al. (17), the apparent group differences in fluorescence intensity observed in Figures 7, 8, and 10 in Ahmed et al. (17) appear to be disproportionately influenced by two extreme outlier values in the non-stimulated group. The study does not clarify whether data were averaged per animal, per image, or per cell, further obscuring the interpretation of these results. Given the small sample sizes and presence of outliers, normality testing is warranted, and non-parametric alternatives to the paired *t*-test should be considered. Notably, the *p*-value for hSOD1 intensity in Figure 10H in Ahmed et al. (17) is omitted and appears to be entirely driven by the same two outliers. Additionally, the caption claims inclusion of wild-type reference images, yet none are presented.

In sum, the immunohistochemical analyses lack the methodological transparency, statistical rigor, and experimental controls necessary to support the claim of a treatment-induced effect.

## 3 Conclusion

ALS has been adversely impacted by a history of failed clinical trials built upon preclinical studies that lacked sufficient rigor. Upholding the highest standards of methodological rigor and adherence to established statistical and scientific practices in preclinical research is not only essential for advancing therapeutic discovery but also an ethical responsibility to prevent the propagation of false hope among patients with ALS.

Yet, the Ahmed et al. study misrepresents survival by using symptom-based metrics and lacks basic scientific rigor, making its claims of extended survival, improved motor function, and reduced disease markers unreliable. Superseding these concerns of rigor, the advertisement of 74% survival improvement is misleading and inaccurate, when the survival was only extended by 5% (7 days). We, therefore, urge the authors to fundamentally consider revising their analysis and their reporting of treatment effects, as the current approach does not allow for a reliable assessment of the true therapeutic potential of their stimulation approach. Without such revisions, the conclusions presented are misleading and overstate the efficacy of the intervention, a concern that is particularly serious in light of the first author's commercial interest in promoting this approach.

To advance the development of more effective treatments for ALS, preclinical studies must prioritize (1) validation of symptom onset and disease progression metrics relative to prior work; (2) proper survival statistical analysis (e.g., log-rank test, Cox models) and transparent reporting of survival data; and (3) rigorous stereological practices, detailed methodological descriptions, and robust statistical approaches for histological assessments. Without these safeguards, we risk perpetuating false hope, diverting attention from more promising approaches, or prematurely dismissing potentially promising therapies.
